# *Wolbachia* infection facilitates adaptive increase in male egg size in response to environmental changes

**DOI:** 10.1038/s41598-025-96680-6

**Published:** 2025-04-16

**Authors:** Eloïse Leroy, Siyi Gao, Maya Gonzalez, Marie-Pierre Ellies-Oury, Midori Tuda

**Affiliations:** 1 Laboratory of Insect Natural Enemies, Faculty of Agriculture, Kyushu University, Fukuoka, 819-0395 Japan; 2 Bordeaux Sciences Agro, 33170 Gradignan, France; 3 Institute of Biological Control, Faculty of Agriculture, Kyushu University, Fukuoka, 819-0395 Japan

**Keywords:** Inclusive fitness, Insect, Modified atmosphere, Parent age, Plasticity, Reproduction, Sex difference, Thermal stress, Evolutionary ecology, Microbial ecology

## Abstract

Under challenging conditions such as maladapted biotic and abiotic conditions, females can plastically adjust their egg size (gamete or zygote size) to counteract fitness declines early in life. Recent evidence suggests that endosymbionts may enhance this egg-size plasticity. Possible endosymbionts’ modification of impact of multiple stressors is not well explored. Therefore, this study aims to test (1) whether *Wolbachia* infection influences the plasticity of parental investment in egg size under suboptimal environmental conditions and (2) whether the plasticity depends on the sex of eggs. We used three lines of the azuki bean beetle (*Callosobruchus chinensis*): a line coinfected with the wBruCon and wBruOri *Wolbachia* strains, a cured line infected solely with the wBruCon, and an uninfected (cured) line. These lines were subjected to either a control environment or a simulated climate change environment (elevated temperature and carbon dioxide levels, eT&CO_2_) to examine *Wolbachia* infection effects on parental investment in their offspring (egg size) and its subsequent impact on offspring fitness, including survival, development, and adult lifespan under starvation. After two days of eT&CO_2_ exposure, coinfected parents increased male egg size only. Larger eggs developed faster in both sexes and exhibited higher survival. However, offspring adult lifespan was not influenced by egg size but by environment, sex, *Wolbachia* infection, and development time: eT&CO_2_ reduced male lifespan but not female lifespan, the singly-infected line females lived longer than coinfected and uninfected line females, and shorter development time linked to longer lifespan. The negative correlation between development time and lifespan was higher under eT&CO_2_ but not sex-specific. This study is the first to demonstrate sex-specific egg size plasticity associated with *Wolbachia* infection in species with sex determination systems other than haplodiploid.

## Introduction

The anthropogenic combustion of fossil fuel steadily increases atmospheric carbon dioxide (CO_2_) concentration, contributing global climate change. By 2100, the average global surface temperature is projected to rise by 2 °C due to the greenhouse effects of CO_2_^1^. Organisms on earth must adapt to these simultaneous changes in multiple environmental factors by plastically or constitutively altering their life history traits, morphology, and behavior to mitigate fitness reductions. In animals, including arthropods, this plasticity can manifest as an increase in zygote (egg) size, which can enhance offspring fitness under challenging conditions^2–5^ (but not necessarily^6^). Such parental investment involves the transfer of maternal resources to eggs, a process influenced by environmental factors like temperature and CO_2_ levels separately^7–10^ or in combination^11^. Additionally, investment strategies may vary with egg sex, as maternal and paternal resources and genetic or epigenetic components can affect offspring traits, including body size, in response to environmental cues, including temperature^12–16^.

*Wolbachia pipientis* (alpha-Proteobacteria) is an endosymbiotic bacterium infecting approximately 52% of terrestrial arthropods^17^. Depending on the host context, *Wolbachia* may establish mutualistic, parasitic, or commensal relationships with their host^18^. These bacteria are maternally transmitted through eggs and are known to induce reproductive manipulations such as cytoplasmic incompatibility (CI), parthenogenesis, feminization, and male-killing in arthropod hosts^18^. CI is the most common and has been reported in various host–*Wolbachia* interactions. CI (or unidirectional CI) results in the death of eggs at early developmental stages that are produced by uninfected females mated with infected males. Since infected females are compatible with both infected and uninfected males, they receive a reproductive advantage compared to uninfected females. In addition, *Wolbachia* influence host reproduction^19,20^, immunity^16,21–23^, behavior^24^, and other fitness traits^25,26^. Fitness effects of *Wolbachia* are context-dependent: Environmental temperature^27,28^, host genotype^29,30^, *Wolbachia* genotype^27,31^, coinfection^23^, and host sex^27,32^ have been shown to influence *Wolbachia* effects on host fitness.

A seed predator, the azuki bean beetle *Callosobruchus chinensis* (Coleoptera: Chrysomelidae: Bruchinae), is one of the most serious pests of stored pulses^33^, laying eggs on fabaceous seeds, where larvae feed and develop within the seeds^34^. Approximately 96% of natural populations are coinfected by two *Wolbachia* strains—wBruCon (referred to as Con hereafter) and wBruOri (referred to as Ori)—while single infections are rare (1.6% with Con, 2.4% with Ori^35^). (Note that the identifiers Con and Ori do not represent phylogenetic groups since they are based on the sequence of *wsp* gene, which exhibits a high recombination rate.) The third “strain” wBruAus was later found to be not a bacterium but a fragmented and pseudogenized *Wolbachia*-like genome remnant transferred to the host sex chromosome, X^36,37^. Coinfection can reduce or increase total *Wolbachia* density compared to single infection with Con^38,39^. Both strains belong to supergroup B^40^ but differ in their infection dynamics, with Ori being more abundant during the egg stage and Con becoming dominant in adults^41^. While *Wolbachia* reduces beetle fecundity, it confers benefits such as shorter development time, higher locomotion, increased number of sperms donated to females per mating^25^. Interestingly, the genome of *C. chinensis* is approximately half the size of that of its relative *C. maculatus*, which is typically uninfected by *Wolbachia*^35,42^.

Arthropod females can adaptively increase egg size in response to suboptimal conditions, such as maladapted host plants^2^ or elevated temperatures^15,43,44^. For a seed beetle, this adjustment requires about two days^2^. Endosymbiotic *Wolbachia* can enhance host tolerance to heat stress^31^. However, evidence of endosymbionts affecting egg size is limited (to haplodiploid arthropod hosts^45,46^). The combined effects of temperatures and CO_2_ on egg size have rarely been tested (but see^11^), let alone the effect that *Wolbachia* infection can have on host egg size response to such environmental changes.

Previous studies have shown that the fecundity, hatchability and carrying capacity of coinfected *C. chinensis* decline with a temperature rise by 2 °C^47–49^. Body size decreased when reared under elevated temperature and CO_2_ (eT&CO_2_) and in coinfected hosts compared to singly-infected hosts regardless of sexes^39^. This study aimed to test (1) whether *Wolbachia* infection influences the plasticity of parental investment in egg size under climate change conditions, eT&CO_2_, suboptimal for *C. chinensis* and (2) whether the plasticity depends on the sex of eggs. We used three lines of *C. chinensis* with different *Wolbachia* infection statuses: a coinfected line (Con and Ori), a singly-infected line (Con only), and an uninfected line. Each line was exposed to either a control environment or a simulated climate change environment. We assessed the impact of *Wolbachia* on parental investment (egg size) and offspring fitness (survival, development time, and lifespan) under these conditions.

## Materials and methods

### Biological materials

It takes 22 days for *C. chinensis* females and 21 days for males to develop from eggs to adult emergence at 30 °C. Females live for approximately 7 days when paired with a single male and oviposit, and males live for approximately 8 days when paired with a single female. Eggs laid by females hatch within 4–5 days. Laboratory cultures of *C. chinensis* (jC strain) were maintained under constant conditions without providing water or food to the adults (aphagously) [30 °C, 60–70% relative humidity (r.h.)] for over 70 years, corresponding to over 1100 generations^50^. An isofemale line was established by randomly selecting a female–male pair from the culture. To create sublines with distinct infection statuses, adults from multiple sublines were treated for one generation with 800 μl of either 0.05% or 0.25% tetracycline solution applied to a filter paper to eliminate one or both strains of *Wolbachia*^39^. This resulted in the establishment of three sublines: coinfected, singly infected (Con only), and uninfected.

### Experimental procedure

**Fig. 1:**
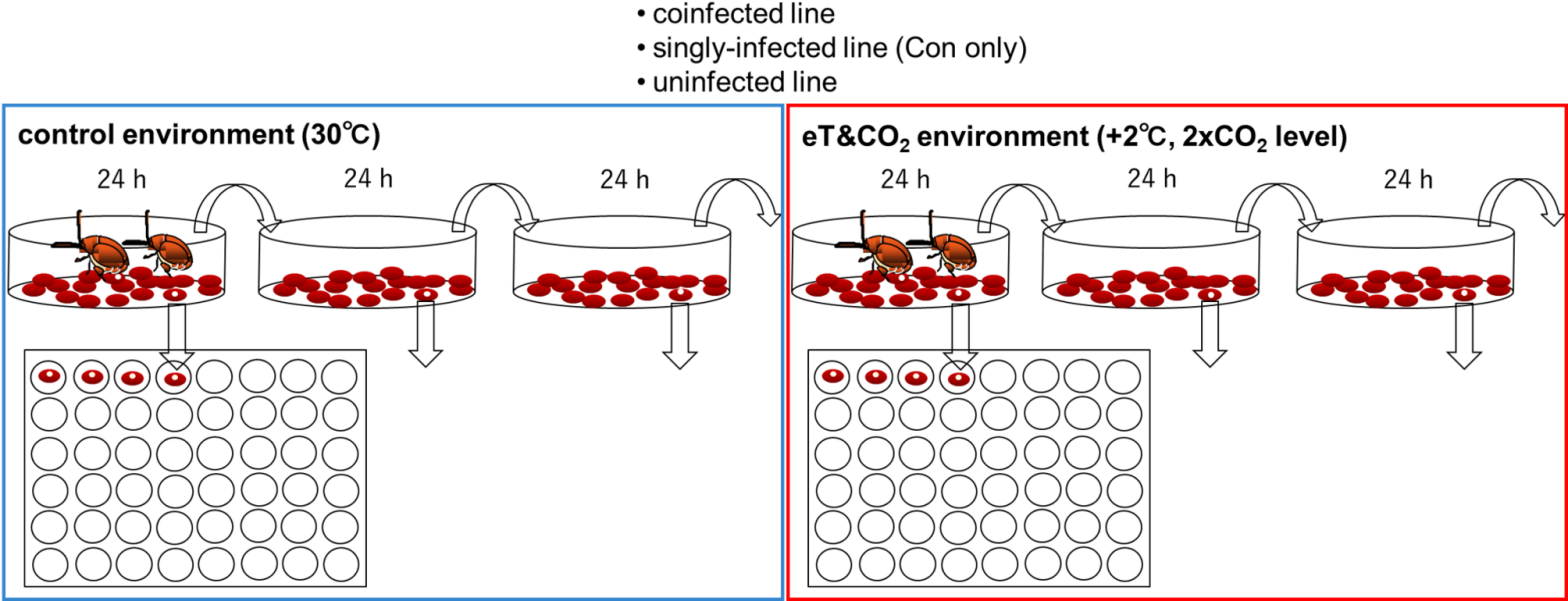
Experiment design for studying egg size plasticity. A pair of beetles from coinfected, singly-infected (Con only), or uninfected lines was allowed to oviposit for three consecutive days under either control conditions or elevated temperature and CO_2_ (eT&CO_2_) conditions (eight pairs for each combination of line and environmental condition). Beans with a single hatched egg were isolated to track the timing of adult emergence and mortality. Beetle size relative to bean size is smaller.

Each *C. chinensis* line was reared at low larval densities (one or two hatched eggs per azuki bean seed, *Vigna angularis* var. *angularis*) to minimize the effects of larval crowding. Newly emerged adults from beans (within 4 h) were collected, and one female–male pair was introduced into a Petri dish containing 5 g of azuki beans. The pairs were allowed to lay eggs under either the control conditions (30 °C, 500–600 ppm CO_2_) or elevated temperature and CO_2_ conditions (32 °C, 1000–1200 ppm CO_2_, referred to as eT&CO_2_), with 60% r.h. and a 16:8 h light–dark cycle. After 24 h, each pair was transferred to a new dish with the same amount of beans. This procedure was repeated three times over 72 h (Fig. 1), and the pair was removed from the third dish. Total of 48 parent pairs were prepared [2 environmental conditions × 3 host lines (*Wolbachia* infection statuses) × 8 parent replicates].

To assess the relationship between egg size (laid by females from different lines on various dates under distinct environments) and survival during the larval/pupal stages, as well as adult lifespan under starvation, beans with a single hatched egg were isolated individually in wells of a 48-well plate and covered with a transparent lid (Fig. 1), one week after the removal of parental pairs. Up to 10 beans per oviposition day per beetle pair were assessed. A single azuki bean seed is sufficient to rear a few *C. chinensis* individuals to adulthood^47^. The length of the eggs (as attached to bean surface) was measured to a precision of 1 μm using a microscope (H-5500, Keyence, Osaka, Japan) at 50 × magnification, before adult emergence started. Starting 19 days after oviposition, adult emergence from each bean was recorded every 24 h, and the sex of the emerged offspring adults was determined based on distinct sex dimorphism in antennal morphology. The death of each offspring adult was subsequently monitored every 24 h until all individuals in the wells had died.

### Statistical analyses

Egg size (length) was analyzed using a general linear mixed model (GLMM) with environment, beetle line (*Wolbachia* infection status), parents’ age, sex, their interactions up to three-way, as fixed effects, and parent ID as a random effect, using the restricted maximum likelihood (REML) method. Logistic regression was applied to test the survival (binary) of offspring from egg hatching to adult emergence, with environment, beetle line, parents’ age, their interactions, egg length, and parent ID as fixed effects, and Bonferroni correction was applied in posthoc pairwise comparisons. Development time (from egg to adult) was log-transformed and tested using GLMM with environment, beetle line, parents’ age, sex, their interactions up to three-way, egg length, as fixed effects, and parent ID as a random effect, using REML. In these analyses, interaction terms were excluded from the highest interaction if its *p* > 0.05. Offspring adult lifespan under starvation was log-transformed and tested using GLMM with environment, beetle line, parents’ age, sex, egg length, and log(development time), as fixed effects, and parent ID as a random effect, using REML. Development time was included for a correlation between these life history traits. For significant variables, their two-way and three-way interactions were also included but non-significant interactions were excluded one by one. In GLMMs, the normality of residual error distributions was verified. For posthoc multiple comparisons, Tukey tests were performed. These tests were conducted for each beetle line separately when complex interaction terms with beetle line were present. Significance level was set at *p* = 0.05. All analyses were conducted using JMP 14.3.0.

## Results

### Egg size plasticity under climate change conditions

**Fig. 2:**
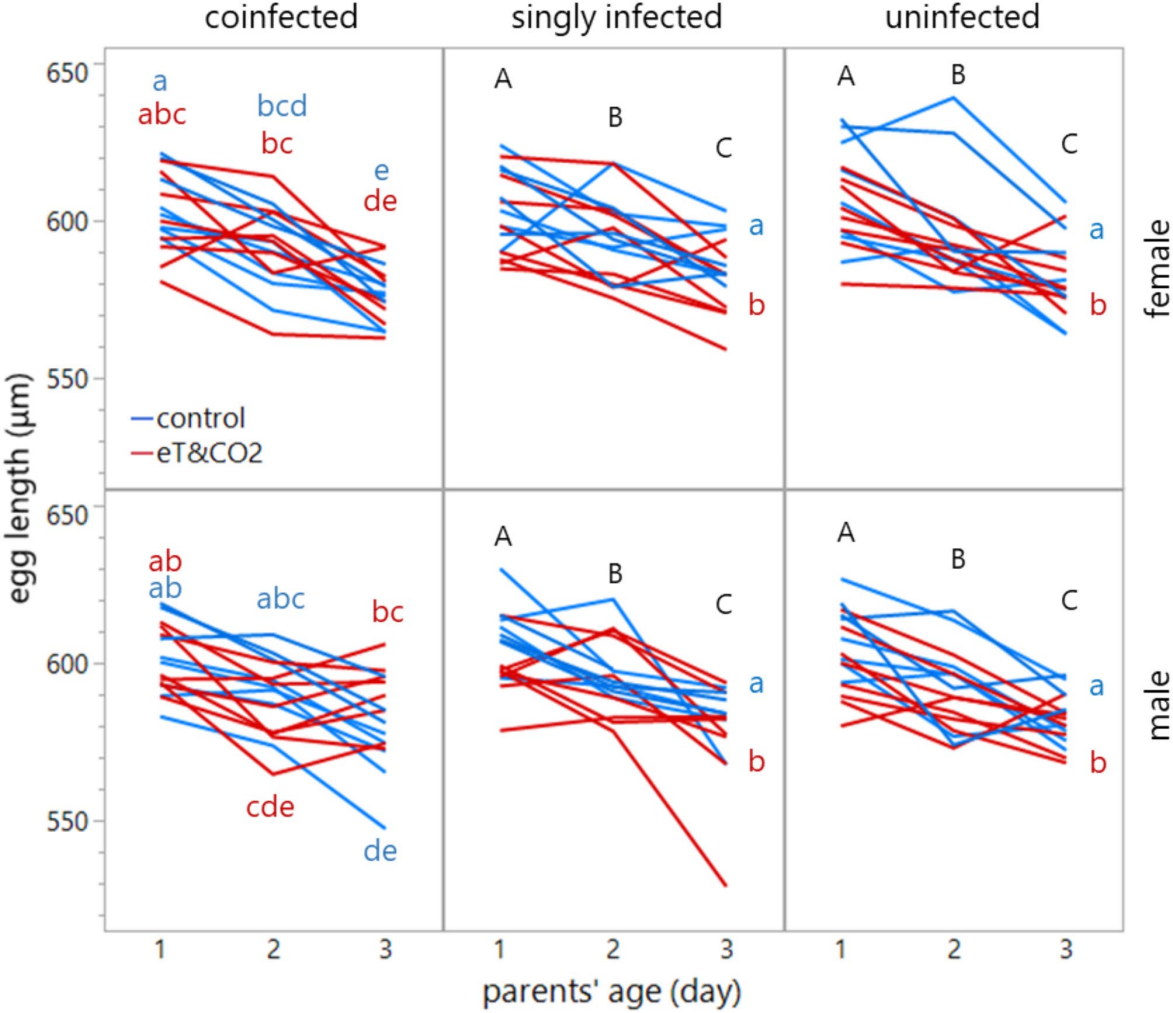
Effects of infection status on egg size across environments, parents’ ages (or parents’ exposure times) and sexes. Mean egg size (with 95% confidence intervals) is shown for coinfected, singly-infected (Con only), and uninfected lines in control environment (in blue) or elevated temperature and CO_2_ (eT&CO_2_) (in red). Different alphabets indicate significant differences within beetle lines.

Egg length for both sexes was significantly influenced by the environment (*p* = 0.049), parents’ age (*p* < 0.001), the interaction between the environment and parents’ age (*p* = 0.004), and three-way interactions among environment, line, and parents’ age (*p* = 0.024) and among environment, parents’ age, and sex (marginally significant, *p* = 0.054; Table 1). Specifically, egg size was reduced under eT&CO_2_ and as parents aged (Fig. 2). Furthermore, the size of male eggs laid by coinfected parents on the third day was larger under eT&CO_2_ compared to under control environmental conditions (Fig. 2). Parent ID as a random effect was significant (*p* < 0.001).

**Table 1:** Egg length. Effect of environment (control or eT&CO_2_), infection status [co-, singly- (Con only), or un-infected], and parents’ age.

	*df1*	*df2*	*F*	*p*
Environment	1	42.14	4.11	**0.049**
Line	2	42.14	0.54	0.586
Parents’ age	2	1304.85	229.87	** < 0.001**
Sex	1	1310.28	0.00	0.944
Environment × line	2	42.12	1.31	0.279
Environment × parents’ age	2	1304.81	5.56	**0.004**
Environment × sex	1	1310.36	0.28	0.599
Line × parents’ age	4	1304.77	0.86	0.489
Line × sex	2	1310.43	0.37	0.690
Parents’ age × sex	2	1311.55	0.90	0.408
Environment × line × parents’ age	4	1304.74	2.81	**0.024**
Environment × parents’ age × sex	2	1311.60	2.93	*0.054*
				Wald *p*
Parent ID				** < 0.001**

General linear mixed model with parent ID as a random effect. Bold *p* values indicate statistical significance. An italicized *p* value indicates marginal statistical significance.

### Survival from hatched eggs to the adult stage

**Fig. 3:**
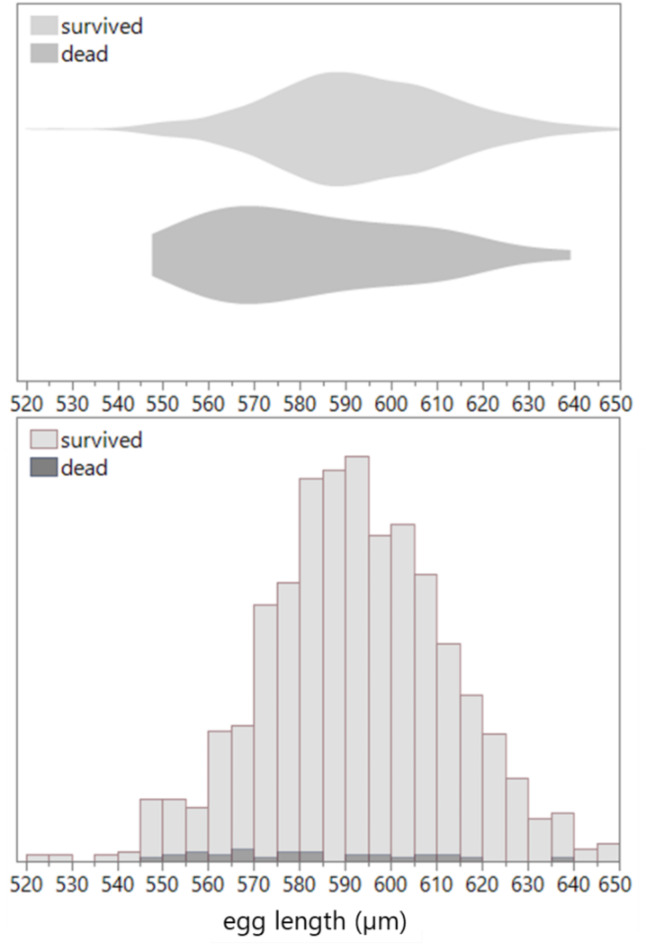
Size distributions (upper panel: data density; lower panel: frequency) of hatched eggs that developed into adults (“survived”) and those that did not survive to adulthood (“dead”).

The sex of the dead individuals in most cases was unable to be determined because of their early developmental stages. Therefore, the data for all eggs was not separated into each sex. Egg size significantly affected survival from hatched eggs to the adult stage (*p* = 0.014; Table 2): Larger eggs have a higher probability of survival (Fig. 3). The effects of environment, infection, and parents’ age, and their interaction effects were not significant (Table 2). Parent ID did not affect survival to the adult (Table 2).

**Table 2:** Probability of adult offspring emergence (survival from hatched eggs to adulthood).

	*df*	${\chi }^{2}$	*p*
Environment	1	0.00	1.000
Line	2	0.00	1.000
Parents’ age	2	1.21	0.546
Egg length	1	6.82	**0.014**
Parent ID	44	50.47	0.233

Logistic regression model. ${\chi }^{2}$: likelihood ratio ${\chi }^{2}$. A significant value is in bold.

### Development time

**Fig. 4:**
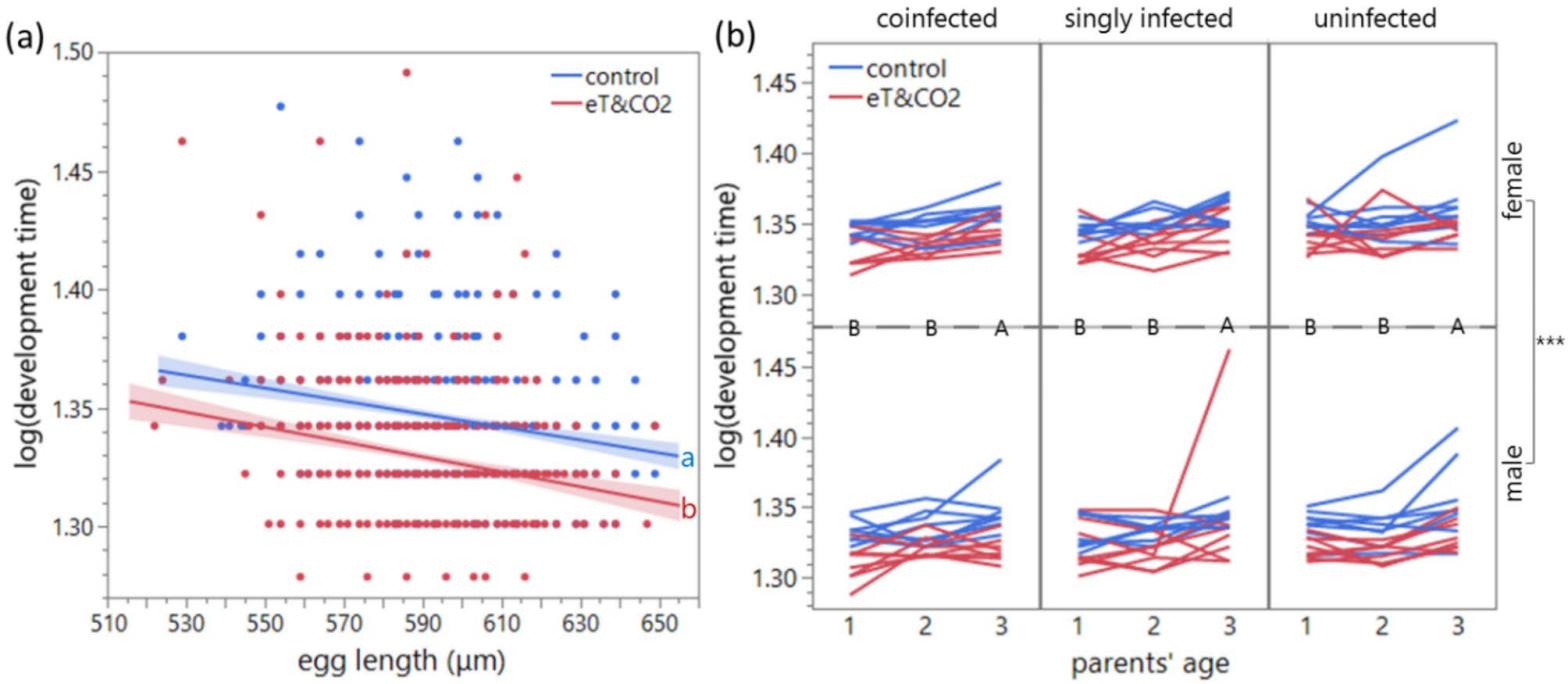
Development time (log-transformed) from egg to adult reared in control environment or eT&CO_2_ (elevated temperature and CO_2_) environment. (**a**) Effect of egg length. Solid lines represent linear regression fits, with shaded bands indicating 95% confidence intervals. (**b**) Effects of parents’ age and offspring sex. Offspring (mean) from same parents relate to solid lines. Effect of beetle line was non-significant. Different alphabets indicate significant differences.

Environment, parents’ age, offspring sex, and egg length significantly affected offspring development time (*p* < 0.001) but beetle line effect was absent (*p* = 0.167) (Table 3): Specifically, eT&CO_2_ reduced development time, offspring from eggs laid on the first and second days developed faster than offspring from the third day eggs, male offspring developed faster, and larger eggs developed faster, regardless of sex and environment (Fig. 4). None of interaction effects were significant and excluded from the model. Parent ID as a random effect was significant (*p* < 0.001).

**Table 3:** Development time from egg to adult (log-transformed).

	*df1*	*df2*	*F*	*p*
Environment	1	44.25	45.37	** < 0.001**
Line	2	43.92	1.87	0.167
Parents’ age	2	1331	18.81	** < 0.001**
Sex	1	1326	184.50	** < 0.001**
Egg length	1	1324	35.58	** < 0.001**
				Wald *p*
Parent ID				** < 0.001**

General linear mixed model with parent ID as a random effect. Statistically significant *p* values are shown in bold.

### Adult lifespan under starvation

**Fig. 5:**
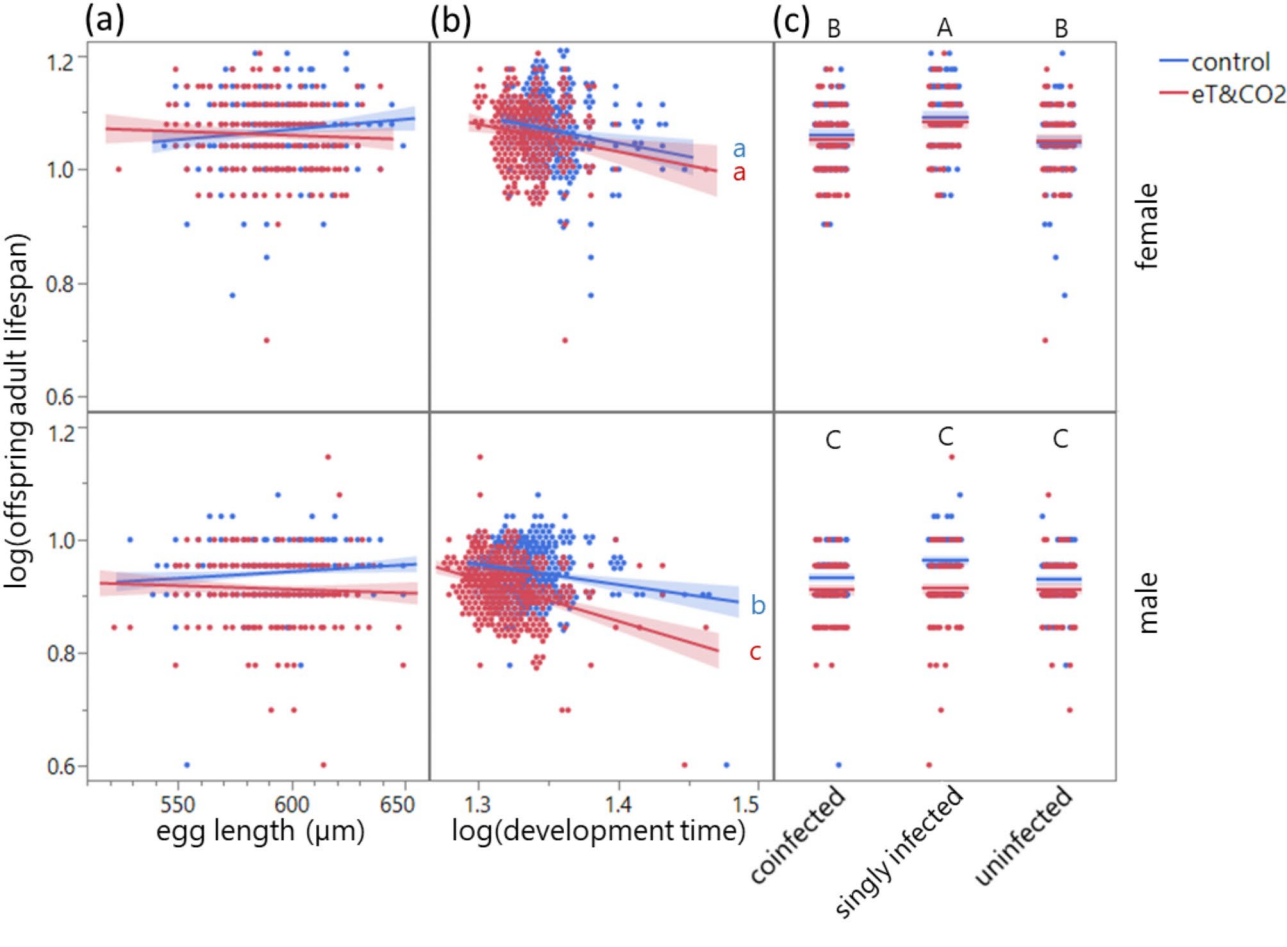
(**a**) Lifespan (log_10_-transformed)(under starvation) of offspring adults developed from eggs laid in different environments. Egg length did not affect lifespan under starvation (*p* = 0.400). (**b**) Correlation of log_10_(offspring adult lifespan under starvation) with log_10_(development time) (*p* < 0.001) and two-way interaction effects on log_10_(offspring adult lifespan under starvation) [environment × sex *p* < 0.001; environment × log_10_(development time) *p* = 0.048]. (**c**) Female and male offspring adult lifespan of beetle lines with different *Wolbachia* infection statuses [coinfected, singly-infected (Con only), and uninfected] laid and reared in control or eT&CO_2_ (elevated temperature and CO_2_) environment (line × sex *p* = 0.008). Different alphabets indicate significant differences. Jittering in (b,c) for visibility.

Environment, line, sex, log(development time) (*p* < 0.001), two-way interactions between environment and sex (*p* < 0.001), line and sex (*p* = 0.008), and between environment and log(development time) (*p* = 0.048) affected offspring adult lifespan (under starvation) but egg size (*p* = 0.400) and parents’ age (*p* = 0.831) did not (Table 4; Fig. 5): Specifically, male offspring lifespan (starvation resistance) from parents exposed to and reared under eT&CO_2_ was shorter but female offspring lifespan was not affected by environments, and females lived longer than males (Fig. 5). Offspring that developed faster lived longer and this negative correlation was stronger under eT&CO_2_, regardless of sex (Fig. 5). The singly-infected (Con only) line female offspring lived longer than co-infected and uninfected line females but no between-line difference was found in male offspring lifespan (Fig. 5). There was no significant two-way interaction effect between sex and log(development time) (*p* = 0.821) and three-way interaction effect among environment, sex, and log(development time) (*p* = 0.257), which were therefore excluded from the model for offspring adult lifespan. Parent ID as a random effect was also significant (*p* = 0.001).

**Table 4:** Adult offspring lifespan under starvation of three beetle lines with different *Wolbachia* infection status in control and eT&CO_2_ (elevated temperature and CO_2_) environments.

	*df1*	*df2*	*F*	*p*
Environment	1	48.97	28.03	** < 0.001**
Line	2	44.11	11.58	** < 0.001**
Parents’ age	2	1265.74	0.19	0.831
Sex	1	1264.56	2449.29	** < 0.001**
Log(development time)	1	1272.48	74.69	** < 0.001**
Egg length	1	1201.27	0.71	0.400
Environment × sex	1	1262.23	21.85	** < 0.001**
Environment × log(development time)	1	1273.14	3.91	**0.048**
Line × sex	2	1257.55	4.81	**0.008**
				Wald *p*
Parent ID				**0.001**

General linear mixed model with parent ID as a random effect. Statistically significant *p* values are shown in bold.

## Discussion

Egg size in *C. chinensis* was influenced by environmental changes and parental age. Exposure to and rearing under elevated temperature and CO_2_ (eT&CO_2_) reduced egg size, accelerated development (regardless of offspring sex), and shortened the lifespan of male offspring adults. Large egg size increased larva-to-adult survival and accelerated development. As previously reported^51^, egg size decreased with parental age, except for the coinfected line. In this line, male egg size increased after two days of eT&CO_2_ exposure compared to control conditions (Fig. 2). Egg size was the primary trait affecting larva-to-adult survival and development time (Figs. 3 and 4), with larger eggs improved both traits. Interestingly, parents selectively enhanced this early-life fitness of male offspring (sons) by increasing male egg size. Faster development, rather than egg size itself, correlated with longer lifespan under starvation in both sexes, particularly under eT&CO_2_ (Fig. 5).

Coinfection with *Wolbachia* strains Con and Ori enhanced the host’s ability to plastically respond to environmental changes by modulating egg size. This response could involve *Wolbachia*’s role in host oogenesis and nutritional provisioning^52–54^. Interestingly, the delay in egg size increase (approximately two days) aligns with the time required for beetle species to adjust to maladaptive hosts^2^ or high temperatures^15^, although a shorter response period has been observed in *C. maculatus* under temperature stress^44^. While examples of endosymbionts influencing host egg size are rare, some exceptions exist in haplodiploid thrips and mites^45,46^. The present study is the first to demonstrate sex-dependent egg size plasticity in a species with sex chromosome-based sex determination (XY male). From a population genetics perspective, stress-induced fitness variation in males can efficiently purge deleterious mutations with minimal demographic cost. In *C. maculatus*, male genetic load (inbreeding depression measured as lifespan) at adult stage is lower than female load at moderate to high temperatures, potentially supporting this view^55^.

Strikingly, the increase in egg size under eT&CO_2_ occurred only in male eggs (Fig. 2). Larger eggs developed faster regardless of sex (Fig. 4), consistent with previous findings in this species^3^ and in *C. maculatus*^5^. Adults that developed faster also lived longer, and this correlation was stronger under eT&CO_2_, regardless of sex. The male-biased increase in egg size may be explained by sexual conflict^56^. Earlier male maturation enhances mating opportunities with newly emerging females, whereas earlier-maturing females might suffer from costs associated with sexual harassment by males^57,58^, despite the potential reproductive advantages of larger females^59^. (Note, however, multiple mating effect on females can also be positive, depending on populations^60^). This could drive parents to prioritize male offspring under stressful conditions to maximize inclusive fitness.

The observed increase in egg size under eT&CO_2_ may not be solely due to high temperature but could also be influenced by elevated CO_2_ or their synergistic effects. Elevated CO_2_ has been shown to increase egg size in the moth *Lobesia botrana*^10^, though this effect may be mediated by plant responses to CO_2_ (increased content of sugar and phenolic compounds) as the plant was grown and used as a larval diet^10^. When reared under combined high temperature and CO_2_ stress, the seed beetle *Zabrotes subfasciatus* exhibited increased body size and protein content but reduced lipid content, although egg size change was not studied^11^. While this study as well as ours could not isolate the independent effects of these abiotic factors, understanding their combined impact is crucial for predicting climate change effects on insect populations.

Our study did not exclude the potential contribution of paternal investment to egg traits to better simulate a natural conditions, where males provide females with nutrient-rich seminal fluid along with sperms (together, ejaculates) during mating. Larger ejaculates have been shown to enhance egg size, fecundity, and female lifespan (under water and food deprivation in *C. chinensis*^61^ and *C. maculatus*^60,62,63^). Male reproductive investment can increase under stressful environments: for instance, female propensity to mate increases under water deprivation in *C. maculatus*^63,64^. Under increased temperature and CO_2_ stresses in our study, parental females may have remated more frequently, potentially contributing to the observed increase in egg size.

Finally, *Wolbachia* influenced offspring adult lifespan under starvation. Removal of Ori from the coinfected line (resulting in single infection with Con) increased female lifespan, whereas complete *Wolbachia* removal reduced it (Fig. 5). This suggests that Con is beneficial for the host, while Ori imposes costs, mitigating the benefit provided by Con in coinfected beetles. The impact of *Wolbachia* on host lifespan varies depending on host sex, genotype and the coevolutionary history of *Wolbachia* infections of particular host populations^16,29,30,65^. Environmental stress also affected lifespan under starvation independently of *Wolbachia* infection effects*.* Male lifespan was shorter under eT&CO_2_ conditions, whereas female lifespan was unaffected, indicating that males are more vulnerable to environmental stress under starvation. Similar findings have been reported for *C. maculatus*, where male lifespan is reduced due to faster body mass loss at higher temperatures^66^. Although increased male egg size contributed to their survival and development early in life, continuous exposure to environmental stress likely negates these benefits later in life. Resilience of females to environmental changes may stem from sexual dimorphism in body size: Females accumulate more resources during development and eventually become larger in body size, which increases thermal tolerance^67^. Males, being smaller, intrinsically may be less tolerant to combined environmental and starvation stresses. Male lifespan is also shown to be driven by maternal genetic effects^68^. Additionally, female beetles exhibited lower activity levels, potentially conserving resources more effectively than males. The combined effects of environmental stress and *Wolbachia* infection (coinfection or Ori infection) may have synergistically reduced lifespan, similar to findings in other insects subjected to high temperature and *Wolbachia* infection stress^69^. While many species of Bruchinae beetles, including *C. chinensis*, are adapted to arid and aphagous conditions^70^, starvation remains a key factor influencing their life history traits^71,72^.

## Conclusions

*Wolbachia* coinfection enabled host beetles to plastically adjust male egg size in response to stressful environments, enhancing early-life survival and fitness. This study is the first to demonstrate sex-specific egg size plasticity due to *Wolbachia* infection in a species with sex chromosome-based sex determination. Further research is needed to elucidate the underlying mechanisms driving this sex-dependent plasticity aided by coinfecting *Wolbachia*.

## Data Availability

The data obtained in this study are available on reasonable request from the corresponding author (MT).
